# Plant and Fungal Polysaccharides in Periodontitis Treatment: Diverse Mechanisms and Therapeutic Roles

**DOI:** 10.1155/ijod/7284794

**Published:** 2025-12-18

**Authors:** Fan Wu, Shangyan Li, Jiawen Chen, Yuqin Shen

**Affiliations:** ^1^ School and Hospital of Stomatology, Guangdong Engineering Research Center of Oral Restoration and Reconstruction, Guangzhou Medical University, Guangzhou, 510182, China, gzhmc.edu.cn; ^2^ Department of Periodontology, Stomatological Hospital, School of Stomatology, Guangzhou Medical University, Guangzhou, 510182, China, gzhmc.edu.cn

**Keywords:** fungal polysaccharides, inflammation, periodontitis, plant polysaccharides, tissue regeneration

## Abstract

**Background:**

Periodontitis is a chronic immune‐mediated inflammatory disease characterized by the progressive degradation of periodontal tissues. Conventional therapies, including anti‐inflammatory agents and antibiotics, are limited by systemic adverse effects and increased microbial resistance, highlighting the need for safer, multi‐targeted treatments.

**Objective:**

This review systematically evaluates the therapeutic potential of plant‐ and fungal‐derived polysaccharides in periodontitis treatment. It focuses on their multifaceted biological activities, including anti‐inflammatory, antioxidant, antimicrobial, and osteo‐regenerative effects, and elucidates key molecular mechanisms, such as nuclear factor kappa‐light‐chain‐enhancer of activated B cells (NF‐*κ*B), mitogen‐activated protein kinase (MAPK), Wnt/*β*‐catenin, and BMP‐2/Smad signaling pathways. Additionally, it identifies current research gaps and proposes strategies to facilitate the translational development of polysaccharide‐based therapies.

**Materials and Methods:**

A systematic literature search was performed across PubMed, Scopus, Web of Science, and Google Scholar databases using a combination of Medical Subject Heading (MeSH) terms and keywords, including “periodontitis,” “plant polysaccharides,” “fungal polysaccharides,” “inflammatory factors,” and “bone regeneration.” A total of 104 studies were included after screening for relevance to the therapeutic roles and molecular mechanisms of plant‐ and fungal‐derived polysaccharides in periodontitis. The dataset encompassed both in vitro and in vivo investigations, and narrative synthesis was employed to integrate findings owing to the substantial heterogeneity in study designs, endpoints, and methodological approaches.

**Conclusion:**

Plant‐ and fungal‐derived polysaccharides have substantial potential as adjunct or alternative therapies for the treatment of periodontitis. Their multifunctional activities, including modulation of inflammation, inhibition of pathogenic biofilms, and promotion of periodontal tissue regeneration, are mediated through complex multi‐pathway interactions. Clinical translation remains limited by structural heterogeneity, a lack of standardized preparation protocols, and insufficient in vivo validation. Future research should prioritize elucidating structure–activity relationships, optimizing extraction and formulation methods, and conducting well‐designed clinical trials. Integrating evidence from 104 studies, this review underscores the anti‐inflammatory, antimicrobial, and osteo‐regenerative effects of these polysaccharides and emphasizes their importance in future clinical applications.

## 1. Introduction

Periodontitis, a chronic inflammatory disease caused by plaque biofilms, initiates a cascade of immune‐mediated responses, culminating in progressive tissue destruction. As the leading cause of tooth loss among adults, it not only compromises oral health but also exerts a profound impact on overall physical and mental well‐being [1]. Globally recognized as the sixth most prevalent disease [2, 3], periodontitis is intricately associated with systemic conditions, such as diabetes, cardiovascular diseases, and rheumatoid arthritis, influencing their progression and prognosis [4–8]. Given its high prevalence and far‐reaching health implications, development of effective and safe therapeutic strategies remains a critical research priority. The primary objectives of periodontitis therapy are to control inflammation, prevent disease progression, and facilitate periodontal tissue regeneration [9–11]. Conventional mechanical plaque removal, while fundamental, is limited by anatomical constraints and the risk of microbial recolonization. Adjunctive treatments, including nonsteroidal anti‐inflammatory drugs (NSAIDs) and antibiotics, have been employed to enhance outcomes; however, their widespread use is limited by adverse effects and increasing microbial resistance [12–15].

**Figure 1:**
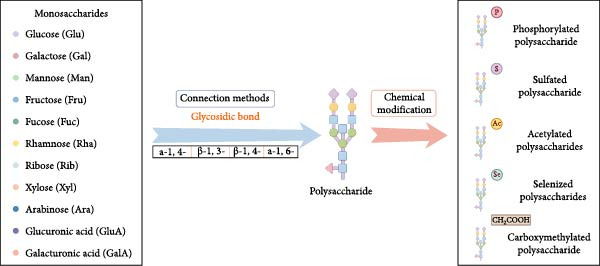
The structure of polysaccharides.

Plant‐ and fungal‐derived polysaccharides are a class of natural macromolecules that have emerged as promising therapeutic candidates. Traditionally, they have been considered as structural components or matrix substances within plants and fungi, with little attention paid to their biological significance. However, recent studies have indicated that their biological activities are far more multifaceted than previously recognized [16]. They not only modulate inflammation but also regulate oxidative stress, influence microbial balance, and promote cellular proliferation, apoptosis, and tissue regeneration. These polysaccharides, extracted from medicinal plants [17] (e.g., *Astragalus membranaceus* and *Lycium barbarum*) and fungi [18] (e.g., *Ganoderma lucidum* and *Dendrobium* spp.), possess favorable properties such as biocompatibility, biodegradability, water solubility, and low toxicity, making them ideal candidates for therapeutic applications. Structurally, plant‐ and fungal‐derived polysaccharides are linear or branched macromolecules composed of 20–10,000 monosaccharide units, such as glucose, galactose, and mannose. Their structural complexity, determined by molecular weight, branching patterns, glycosidic linkages (*α* or *β*), and functional groups (e.g., sulfate, carboxyl, and acetyl), underlies their diverse physicochemical properties and broad biological activities (Figure 1) [16, 19]. For example, higher branching enhances antiaging effects [14], while sulfate groups strengthen antioxidant and antiviral capabilities [15].

Functionally, plant‐ and fungal‐derived polysaccharides display broad pharmacological activities, including immunomodulatory, antioxidant, anti‐inflammatory, antimicrobial, and antitumor effects. By regulating cytokine production, reducing oxidative stress, and modulating cellular processes such as apoptosis, autophagy, and macrophage polarization, they have demonstrated therapeutic relevance in diverse inflammatory conditions such as liver inflammation [20, 21], pneumonia [22, 23], and inflammatory bowel disease [24, 25]. Representative examples, such as *Astragalus*, *Fructus mori*, *Huaier*, and *Ganoderma lucidum* polysaccharides, exhibit strong anti‐inflammatory activity by downregulating proinflammatory mediators and interfering with bacterial pathogenicity [26–29].

These pleiotropic properties have attracted increasing attention owing to their potential in periodontitis, a disease characterized by chronic inflammation, microbial dysbiosis, and progressive tissue destruction. Recent studies have shown that *Hedysari*, Changbai Mountain *Ganoderma lucidum*, and *Lycium barbarum* polysaccharides (LBPs) can mitigate inflammation, suppress pathogenic biofilm formation, and promote periodontal tissue repair [30–32]. Similarly, polysaccharides from jujube, astragalus, and angelica contribute to microbial homeostasis and periodontal regeneration by modulating oxidative stress, cellular metabolism, proliferation, and apoptosis [33–35].

Despite these promising findings, critical knowledge gaps remain. The precise mechanisms by which polysaccharides interact with periodontal tissues and microbial communities, their structure–activity relationships, and their in vivo efficacy require further elucidation. Therefore, a comprehensive synthesis of the current evidence is urgently needed. This review evaluates recent experimental and preclinical studies; elucidates key molecular mechanisms, including nuclear factor kappa‐light‐chain‐enhancer of activated B cells (NF‐*κ*B), mitogen‐activated protein kinase (MAPK), Wnt/*β*‐catenin, and bone morphogenetic protein 2 (BMP‐2)/Smad pathways; and identifies research priorities to guide the translational development of polysaccharide‐based therapies as adjunctive or alternative strategies for improving periodontitis outcomes.

## 2. Method of Searching the Available Literature

**Figure 2:**
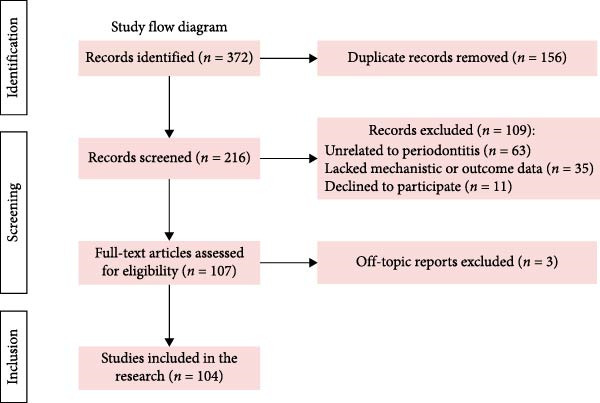
Flowchart for the inclusion and exclusion process.

A systematic literature search was conducted to identify relevant studies from their inception up to 2025 across PubMed, Scopus, Web of Science, and Google Scholar, using a combination of Medical Subject Heading (MeSH) terms and keywords such as “periodontitis,” “plant polysaccharides,” “fungal polysaccharides,” “inflammatory factors,” and “bone regeneration.” A total of 372 records were initially identified through the database search. After removing duplicates, 216 articles remained. The studies were screened based on predefined inclusion and exclusion criteria. The inclusion criteria included studies that investigated plant‐ or fungal‐derived polysaccharides with relevance to periodontitis treatment; reported molecular mechanisms (e.g., NF‐*κ*B, MAPK, and Wnt/*β*‐catenin signaling); and provided either preclinical or clinical evidence related to anti‐inflammatory, antimicrobial, or osteo‐regenerative outcomes. Articles that were unrelated to periodontitis, lacked mechanistic or outcome data, or were conference abstracts without accessible full texts were excluded. After screening, 109 articles were excluded because they did not meet the criteria, and 107 articles were eligible for full‐text review. Finally, 104 studies were included in the narrative review (Figure 2).

Owing to the significant heterogeneity in study designs and the scarcity of clinical trials, a narrative review approach was deemed the most appropriate for synthesizing the findings. This method enabled a detailed examination of the multifaceted roles that polysaccharides play in periodontitis management. Simultaneously, it facilitated the identification of existing research gaps, including the urgent need for standardized polysaccharide preparation protocols and robust clinical validation to support their translation into therapeutic applications.

## 3. Results

### 3.1. Plant and Fungal Polysaccharides in Inflammatory Regulation

During periodontitis progression, the immune system initiates an inflammatory cascade to combat pathogens. However, overactivation of inflammatory mediators inflicts collateral damage on periodontal tissues [36]. Therefore, precise regulation of inflammatory factors is pivotal for effective periodontitis treatment. In recent years, natural plant and fungal polysaccharides have emerged as promising candidates for modulating the dysregulated inflammatory responses in periodontitis. These polysaccharides can attenuate inflammation by regulating immune cell functions (e.g., macrophages) and inhibiting the synthesis and release of proinflammatory cytokines (Table 1). For instance, sulfated polysaccharides from *Turbinaria ornata*, jujube polysaccharides (JPs), and Changbai Mountain *Ganoderma lucidum* polysaccharides have been shown to suppress the production of inflammatory mediators such as interleukin (IL)‐6, tumor necrosis factor‐alpha (TNF‐*α*), inducible nitric oxide synthase (iNOS), and nitric oxide in macrophages [31, 33, 37, 43]. *β*‐glucan, a common structural component in many polysaccharides, also exerts anti‐inflammatory effects by modulating cytokine levels and signaling pathways [44, 45]. Mechanistically, these polysaccharides act through key signaling pathways, most notably the NF‐*κ*B and receptor activator of nuclear factor kappa‐B (RANK)/receptor activator of nuclear factor‐*κ*B ligand (RANKL)/osteoprotegerin (OPG) pathways [38, 39].

**Table 1:** The role of plant and fungi polysaccharides in inflammatory regulation.

Compound name	Sources of polysaccharides	Major monosaccharides	Experimental model	Experimental species	Participation mechanisms	References
Jujube polysaccharide	Jujube	Galacturonic acid, arabinose, galactose, rhamnose, glucose and xylose	LPS‐stimulated and MRSA‐infected macrophages	Homo	Reduce the levels of IL‐1*β*, IL‐6 and TNF‐*α*	[33]
*Ganoderma lucidum* polysaccharide	Changbaishan *ganoderma lucidum*	Glucose, galactose, mannose, xylose, rhamnose, glucuronic acid	Pg‐LPS‐induced RAW264.7 macrophages	Mice	Regulate the contents of inflammatory mediators TNF‐*α*, IL‐1*β*, IL‐10, and NO	[37]
*Lycium barbarum* polysaccharide	*Lycium barbarum*	Rhamnose, arabinose, xylose, mannose, glucose and galactose	LPS‐induced periodontal stem cells	Homo	Inhibit the expression of TNF and IL‐1*β* genes	[32]
Cashew gum polysaccharide	*Anacardium occidentale* L	Galactose, glucose, arabinose, rhamnose, mannose and glucuronic acid	Nylon ligature‐induced periodontitis model	Rats	Inhibit the expression of TNF‐*α* and IL‐1*β*	[38]
Fucoidan	Fucus vesiculosis	L‐fucose and sulfate groups	RAW 264.7 cells	Mice	Inhibit the expression of TNF‐*α* and IL‐6; reduce the levels of iNOS and COX‐2	[39]
*Hedysari* polysaccharide	*Radix hedysari*	Rhamnose, arabinose, xylose, glucose and galactose	LPS‐induced periodontal cells	Homo	Inhibit the secretion of IL‐1*β* and IL‐6	[30]
*Hedysari* polysaccharide	*Radix hedysari*	Rhamnose, arabinose, xylose, glucose and galactose	LPS‐induced periodontal cells	Homo	Inhibit the secretion of TNF‐*α*	[40]
*Dendrobium officinale* neutral polysaccharide	*Dendrobium officinale*	Rhamnose, mannose, glucose and galactose	Pg LPS‐induced oral keratinocytes	Homo	Inhibit the phosphorylation of the TLR2 protein channel, and decrease the levels of IL‐6, IL‐8 and TNF‐*α*	[41]
*Morinda officinalis* polysaccharide	*Morinda officinalis*	Rhamnose, arabinose, xylose, mannose, glucose, fructose, galactose, galacturonic acid	Inflammatory periodontal ligament cells	Homo	Promoted the expression of SIRT1, resulting in the inhibition of NLRP3; reduce the acetylation level of NLRP3 through deacetylation, thereby decreasing its activity	[42]

The NF‐*κ*B signaling pathway is central to the pathogenesis of periodontitis. Under normal conditions, NF‐*κ*B remains inactive in the cytoplasm, sequestered by its inhibitor I*κ*B. Upon lipopolysaccharide stimulation in periodontal tissues, I*κ*B undergoes phosphorylation and degradation, releasing NF‐*κ*B. The liberated NF‐*κ*B then translocates to the nucleus, where it initiates the transcription of proinflammatory genes [46–49]. Fucoidan, a sulfated polysaccharide, has demonstrated inhibitory effects on the NF‐*κ*B pathway. In vitro, fucoidan reduces the expression of proinflammatory cytokines (TNF‐*α*, IL‐1*β*, and IL‐6), as well as iNOS and cyclooxygenase‐2 proteins, subsequently decreasing the production of nitrite and prostaglandin E2 in macrophages [39]. However, in a *Porphyromonas gingivalis* (*Pg*)‐induced periodontitis mouse model, the efficacy of fucoidan in reducing proinflammatory cytokines and preventing alveolar bone loss was limited. This discrepancy between in vitro and in vivo results underscores the complexity of the anti‐inflammatory actions of fucoidan, which are likely influenced by factors such as bioavailability, metabolic transformation, immune microenvironment, and cell‐type‐specific responses in vivo.

Polysaccharides can also interact directly with toll‐like receptors (TLRs), upstream regulators of the NF‐*κ*B pathway, to modulate immune responses in periodontitis. For example, *Hedysari* polysaccharide (HPS) downregulates the secretion of IL‐1*β*, IL‐6 [30], and TNF‐*α* [40] in periodontal cells via the TLR4/NF‐*κ*B axis, thereby reducing local inflammation. *Dendrobium officinale* polysaccharide (DOP)‐1 inhibits TLR2 activation, decreasing the expression and release of IL‐6, IL‐8, and TNF‐*α*, which in turn mitigates cell apoptosis and periodontal tissue damage [41, 50]. *Morinda officinalis* polysaccharide not only reduces the level of fibronectin extra domain A, an endogenous TLR4 ligand in the periodontal inflammatory milieu but also suppresses the activation of NLRP3 inflammasome, leading to decreased IL‐1*β* and IL‐18 production and reduced inflammatory cell infiltration in periodontitis rats [42, 51, 52]. LBP may exert anti‐inflammatory effects by simultaneously inhibiting NF‐*κ*B and the NLRP3 inflammasome pathways [53, 54].

In addition to cytokine regulation, certain polysaccharides enhance the antioxidant defense system in periodontal cells, thereby indirectly curbing inflammation. Polysaccharides such as fucoidan, JPs, and Changbai Mountain *Ganoderma lucidum* polysaccharides exhibit dual antioxidant and anti‐inflammatory properties by suppressing proinflammatory mediators in macrophages [31, 33, 37, 43, 55]. Their antioxidant mechanisms involve multiple pathways: scavenging free radicals through hemiacetal hydroxyl groups, interrupting lipid peroxidation, chelating pro‐oxidant metal ions (Fe^2+^ and Cu^2+^), and upregulating endogenous antioxidant enzymes (e.g., superoxide dismutase [SOD] and glutathione [GSH]). For instance, sulfated polysaccharides from *Turbinaria ornata* can increase GSH levels by 50% and SOD activity by 100%, highlighting their potent antioxidant potential [43]. Sulfation at specific hydroxyl positions (C2, C4) likely contributes to this activity, although the precise structure‐function relationships remain to be fully elucidated owing to the structural complexity of the polysaccharides.

Polysaccharides also play a role in maintaining the regenerative capacity of periodontal tissues by acting on the periodontal stem cells. LBP, for example, inhibits the expression of TNF and IL‐1*β* in periodontal ligament stem cells (PDLSCs), reduces reactive oxygen species (ROS) levels, and protects cells from oxidative and inflammatory damage [32]. In a rat periodontitis model, cashew gum polysaccharide (CG‐P)‐containing oral gel significantly reduced alveolar bone loss, decreased the mRNA expression of TNF‐*α*, IL‐1*β*, RANKL, and the RANKL/OPG ratio, and lowered myeloperoxidase activity in gingival tissues [38].

Collectively, plant and fungal polysaccharides modulate periodontitis‐associated inflammation via a multidimensional approach that targets multiple cellular and molecular pathways. Their ability to suppress inflammatory mediators, regulate key signaling cascades, enhance antioxidant defenses, and preserve tissue regenerative potential makes them promising candidates for periodontitis therapy. However, several challenges remain, including the need for standardized extraction and purification methods, elucidation of structure–activity relationships, and large‐scale clinical trials to validate their safety and efficacy. Future studies should focus on translating these findings into clinically applicable treatments to offer new therapeutic strategies against this prevalent and debilitating disease.

### 3.2. Regulatory Effects of Plant and Fungal Polysaccharides on Cell Autophagy

Cellular autophagy is a highly conserved catabolic process that exerts a dual‐edged regulatory influence on periodontitis progression [56]. On one hand, it can exacerbate periodontal inflammation by promoting inflammasome activation, specifically the NLRP3 signaling pathway, and upregulating proinflammatory cytokines such as IL‐1*β* and IL‐18. This cascade stimulates osteoclastogenesis and differentiation, accelerating inflammation and alveolar bone resorption. Conversely, autophagy acts as an endogenous anti‐inflammatory mechanism that eliminates damaged organelles and sequesters inflammatory mediators. In periodontitis, host immune‐inflammatory responses often trigger autophagy as a self‐protective mechanism to dampen excessive inflammation, underscoring its complex role in disease pathogenesis.

Recent studies have revealed that plant and fungal polysaccharides, which are characterized by their intricate structural diversity and chemical complexity, modulate autophagy in a bidirectional manner (Table 2). For example, medium and low concentrations of *Astragalus* polysaccharides have been shown to inhibit autophagic flux in periodontal ligament fibroblasts. This inhibition is manifested through multiple mechanisms, including upregulation of the autophagy receptor p62, suppression of the autophagy‐related protein Beclin‐1, blockade of microtubule‐associated protein 1 light chain 3 (LC3) conversion from LC3‐I to LC3‐II, and suppression of the AMP‐activated protein kinase/mammalian target of rapamycin complex 1 signaling pathway. Collectively, these effects reduce autophagy, promote cell proliferation, and inhibit apoptosis, thereby facilitating periodontal tissue repair [57].

**Table 2:** The regulatory effects of plant and fungal polysaccharides on cell autophagy.

Compound name	Sources of polysaccharides	Major monosaccharides	Experimental model	Experimental species	Participation mechanisms	References
*Astragalus* polysaccharide	*Astragalus radix*	Glucose, arabinose, galactose, rhamnose and xylose	Human periodontal fibroblasts	Homo	Regulate AMPK/mTORC1 signaling pathway	[57]
*Agaricus blazei* Murrill polysaccharides	*Agaricus blazei* Murrill	Glucose, arabinose, galactose, galacturonic acid, fucose, rhamnose, mannose	LPS‐induced human periodontal cells and periodontitis rat	Homo, rat	Enhance the H_2_S/NRF2 axis, promote cellular autophagy, and reduce the expression of IL‐1*β*, IL‐6, IL‐8, TNF‐*α*, and iNOS	[58]

In contrast, *Agaricus blazei* Murrill polysaccharide (ABMP), composed of a diverse array of monosaccharides, including glucose, arabinose, galactose, galacturonic acid, fucose, alginate, rhamnose, and mannose, exerts pro‐autophagic effects in periodontal fibroblasts. ABMP activates the hydrogen sulfide/nuclear factor erythroid 2‐related factor 2 signaling axis, leading to enhanced autophagic activity. This upregulation results in a significant reduction in proinflammatory mediators such as IL‐1*β*, IL‐6, IL‐8, TNF‐*α*, and iNOS. By facilitating the clearance of intracellular toxins and suppressing inflammatory cytokine production, ABMP effectively alleviates periodontitis, reduces osteoclast activation, and mitigates alveolar bone loss [58].

Notably, despite their shared goal of improving periodontal health, *Astragalus* polysaccharides and ABMP employ opposing autophagy‐regulatory strategies. While *Astragalus* polysaccharides promote tissue regeneration by suppressing autophagy, ABMP alleviated inflammation by enhancing autophagy. This dichotomy highlights the critical role of polysaccharide structure, particularly monosaccharide composition and functional group configuration, in dictating autophagy‐regulatory outcomes. Future research should focus on deciphering the precise molecular mechanisms underlying these structure‐function relationships, which is essential for optimizing polysaccharide‐based therapies and predicting their therapeutic efficacy in periodontitis treatment.

### 3.3. Antimicrobial Effects of Plant Polysaccharides and Fungi Polysaccharides

The pathogenesis of periodontitis is intricately linked to the formation of plaque biofilms by pathogenic microorganisms, with *Pg* being a pivotal pathogen. Owing to its high virulence, antibiotic resistance, and potent inflammation‐inducing properties, *Pg* secretes an array of virulence factors that drive the initiation and progression of periodontal tissue destruction [59]. Emerging evidence highlights the significant antimicrobial potential of plant‐ and fungal‐derived polysaccharides against these pathogens (Table 3).

**Table 3:** The antimicrobial effects of plant polysaccharides and fungi polysaccharides.

Compound name	Sources of polysaccharides	Major monosaccharides	Experimental model	Experimental species	Participation mechanisms	References
*Rhododendron ferrugineum* L. polysaccharides	*Rhododendron ferrugineum* L.	Arabinose, galactose	Pg‐induced erythrocytes	Homo	Affect the outer membrane proteins of Pg	[60]
*Panax ginseng* acid polysaccharide	*Panax ginseng*	Glucuronic acid, arabinose, galactose and glucose	Pg‐induced erythrocytes	Homo	Inhibit the binding of Pg to host cells	[61]
Jujube polysaccharide	Jujube	Galacturonic acid, arabinose, galactose, rhamnose, glucose and xylose	*S. mutans*, MRSA and Pg	Bacterium	Inhibit the growth and biofilm formation of *S. mutans*, MRSA and Pg; prevent the attachment of *S. mutans* to teeth; inhibit the invasion and cytotoxic effects of Pg on host cells	[33]
Konjac glucomannan	Konjac	Mannose, glucose	Pg	Bacterium	Inhibit the proliferation of Pg and its biofilm	[62]
Fucoidan	Brown algae	L‐fucose and sulfate groups	Actinobacillus actinomycetemcomitans, *Fusobacterium nucleatum*, *Prevotella intermedia* and Porphylomonas gingivalis	Bacterium	May affect bacterial cell wall synthesis	[63]

Bacterial adhesion is a critical early event in biofilm formation and the onset of periodontitis. Pathogens utilize adhesins and pili to adhere to periodontal tissues, thereby establishing biofilms that trigger inflammatory responses and tissue damage. Therefore, strategies targeting bacterial adhesion are essential for the treatment of periodontitis. Several polysaccharides have demonstrated potent anti‐adhesion properties. Polysaccharides derived from *Rhododendron ferrugineum* L. have been shown to modify the outer membrane proteins of *Pg*, thereby reducing its adhesion efficiency by ~75% [60]. Similarly, a uronic acid‐rich polysaccharide extracted from *Panax ginseng* roots inhibits *Pg* binding to host cells with a minimum inhibitory concentration of 0.25 mg/mL [61]. The pectin‐type polysaccharides PG‐F2 and PG‐HMW, also isolated from *Panax ginseng*, exhibit dose‐dependent anti‐adhesion effects against oral pathogens [64]. In contrast, acidic polysaccharides from *Artemisia capillaris* leaves lack comparable anti‐adhesion activity [61]. Structural analysis reveals that the *Panax ginseng* polysaccharide, rich in glucuronic acid, effectively blocks *Pg* adhesion, whereas the *Artemisia capillaris* polysaccharide, characterized by a high galacturonic acid content and the absence of glucuronic acid, fails to achieve similar efficacy. These findings underscore the critical role of the uronic acid type and content in determining the anti‐adhesion potential of polysaccharides.

Following adhesion, pathogenic bacteria rapidly proliferate within biofilms, secreting enzymes and toxins that degrade collagen fibers and other periodontal tissue components. Thus, the inhibition of bacterial growth and biofilm maturation is a key therapeutic target. Numerous plant and fungal polysaccharides have been shown to impede the proliferation of periodontal pathogens through diverse mechanisms. These include interactions with bacterial cell wall glycoprotein receptors, which lead to altered membrane permeability, disrupted nutrient uptake, and growth inhibition [65]. Some polysaccharides also downregulate genes associated with biofilm formation [66]. JP, for instance, not only inhibits *Pg* growth but also prevents biofilm formation and disrupts established biofilms [33]. At a concentration of 2.5 mg/mL, JP reduces bacterial adhesion by 75%, and at 10 mg/mL, JP mitigates *Pg*‐induced HEp‐2 cell damage from 60% to 20%.

Despite its distinct monosaccharide composition compared with that of JP, konjac glucomannan exhibits significant antibacterial activity against *Pg*. It inhibits *Pg* proliferation at rates ranging from 15% to 23% and has bactericidal effects of 11%–20% [62]. These observations indicate that antimicrobial efficacy depends not only on monosaccharide composition but also on molecular weight, glycosidic linkages, and functional groups [67]. For example, increasing the uronic acid and rhamnose contents enhances antibacterial activity [68]. In particular, sulfate groups can modify the bacterial surface charge, disrupt metabolism, interfere with cell wall synthesis, and dismantle biofilm structure [69, 70]. Fucoidan, a sulfated polysaccharide, exhibits broad‐spectrum activity against key periodontal pathogens, likely by interfering with cell wall synthesis. Notably, fucoidan demonstrates synergistic effects when combined with antibiotics such as ampicillin or gentamicin, reducing pathogen MICs by fourfold or more [63].

Plant and fungal polysaccharides exert multifaceted antimicrobial effects in periodontitis by targeting bacterial adhesion, growth, and biofilm formation. Their bioactivity is intricately linked to their chemical structures, including monosaccharide composition, molecular weight, and the presence of functional groups such as uronic acid and sulfates. However, owing to the complex and variable nature of polysaccharide structures, further research is imperative to fully elucidate structure–function relationships. Such knowledge is essential for optimizing polysaccharide‐based antimicrobial therapies and developing targeted treatments for periodontitis.

### 3.4. Regulatory Effects of Plant and Fungal Polysaccharides on Periodontal Cells

In the dysregulated inflammatory microenvironment of periodontitis, periodontal tissue cells undergo a detrimental cascade of pathological events, including oxidative stress, apoptosis, necrosis, and aberrant proliferation and differentiation. Elevated ROS levels disrupt cellular redox homeostasis, precipitating cell death and impeding endogenous repair mechanisms in periodontal tissues [71]. Concurrently, the persistent presence of proinflammatory cytokines dysregulates the normal proliferation and osteogenic differentiation of periodontal stem cells, thereby severely compromising their tissue regenerative capacity.

Accumulating evidence has underscored the therapeutic potential of plant‐ and fungal‐derived polysaccharides in modulating these pathological processes to facilitate periodontal tissue repair. These natural macromolecules exert their effects through a multifaceted approach, prominently involving the regulation of critical signaling pathways such as the WNT signaling cascade and microRNA (miRNA) regulatory networks (Table 4).

**Table 4:** The regulatory effects of plant and fungal polysaccharides on periodontal cells.

Compound name	Sources of polysaccharides	Major monosaccharides	Experimental model	Experimental species	Participation mechanisms	References
*Lycium barbarum* polysaccharide	*Lycium barbarum*	Rhamnose, arabinose, xylose, mannose, glucose, and galactose	LPS‐induced periodontal stem cells	Homo	Improve the viability of LPS‐stimulated periodontal ligament stem cells, down‐regulate the level of ROS	[32]
*Hedysari* polysaccharide	*Radix hedysari*	Rhamnose, arabinose, xylose, glucose, and galactose	LPS‐induced periodontal cells	Homo	Stimulated the cell proliferation	[30]
*Hedysari* polysaccharide	*Radix hedysari*	Rhamnose, arabinose, xylose, glucose, and galactose	LPS‐induced periodontal ligament cells	Homo	Regulate the Wnt signaling pathway; reduce the levels of TNF‐*α* and ROS	[40]
*Angelica* polysaccharide	*Angelica sinensis*	Arabinose, galactose, and glucose	Periodontal ligament stem cells	Homo	Upregulate the expression of miR‐301a‐5p	[35]

LBP demonstrates this therapeutic potential. By effectively scavenging excess ROS within the inflammatory milieu, LBP mitigates oxidative damage, thereby enhancing the survival and functionality of PDLSCs and promoting tissue repair [32]. HPS, despite sharing a similar monosaccharide composition with LBP, exhibits unique regulatory mechanisms. HPS activates the WNT signaling pathway, suppresses the secretion of TNF‐*α*, reduces intracellular ROS accumulation, inhibits apoptosis, and stimulates the proliferation and osteogenic differentiation of human periodontal ligament cells [30, 40]. Intriguingly, among its distinct fractions, HPS‐1 demonstrates superior osteogenic potential compared with HPS‐2 and HPS‐3. This enhanced activity can be attributed to its specific structural features, including *α*‐glycosidic linkages, a high glucose content (87.41%), and a lower weight‐average molecular weight (9.336 × 10^3^ g/mol) [72]. These findings highlight the critical role of polysaccharide structural characteristics, such as monosaccharide composition, molecular size, and glycosidic configuration, in determining their biological activities. Although LBP and HPS share functional similarities, subtle differences in their compositional ratios and structural arrangements likely underlie their distinct regulatory profiles.

In addition to canonical signaling pathways, certain plant polysaccharides exert reparative effects by modulating miRNA networks. For instance, *Angelica* polysaccharide has been shown to reduce periodontal ligament cell apoptosis and promote cell viability and proliferation by upregulating miR‐301a‐5p. This miRNA upregulation increases the expression of the anti‐apoptotic protein Bcl‐2 while decreasing levels of the pro‐apoptotic protein Bax [35], effectively tipping the cellular balance towards survival and tissue regeneration.

In summary, plant and fungal polysaccharides are promising therapeutic agents for the management of periodontitis, acting through multiple mechanisms to alleviate oxidative stress, inhibit apoptosis, and promote cell proliferation and osteogenic differentiation. However, the complex nature of these polysaccharides requires further in‐depth investigation to identify their bioactive components and fully elucidate the underlying molecular mechanisms. This research will be instrumental in translating these natural compounds into effective clinical therapies for periodontal disease.

### 3.5. Plant and Fungal Polysaccharides in Inhibiting Bone Resorption and Promoting Bone Formation

Recent studies have delved deeper into the molecular mechanisms by which plant‐ and fungus‐derived polysaccharides regulate periodontal bone remodeling. These polysaccharides do not merely function as anti‐inflammatory agents; instead, they are intricately involved in complex signaling pathways that govern osteoclast activity, shape the immune microenvironment, and stimulate osteogenic differentiation (Table 5). Their diverse biological activities range from immunomodulation to the promotion of cellular processes crucial for periodontal tissue regeneration.

**Table 5:** The role of plant and fungal polysaccharides in inhibiting bone resorption and promoting bone formation.

Compound name	Sources of polysaccharides	Major monosaccharides	Experimental model	Experimental species	Participation mechanisms	References
*Ganoderma lucidum* polysaccharide	Changbaishan *ganoderma lucidum*	Glucose, galactose, mannose, xylose, glucuronic acid, fucose, rhamnose, galacturonic acid, and arabinose	Periodontitis mice	Mice	Inhibited alveolar bone resorption	[31]
*Ganoderma lucidum* polysaccharide	Changbaishan *ganoderma lucidum*	Glucose, galactose, mannose, xylose, rhamnose, glucuronic acid	Pg‐LPS‐induced RAW264.7 macrophages	Mice	Inhibited alveolar bone resorption	[37]
*Armillaria mellea* polysaccharide	*Armillaria mellea*	Glucose, galactose, mannose, fucose, ribose, xylose, glucuronic acid, arabinose, galacturonic acid	Periodontitis mice	Mice	Inhibited alveolar bone resorption	[73]
Konjac glucomannan	Konjac	Mannose, glucose	Periodontitis mice	Mice	Inhibited alveolar bone resorption	[62]
*Astragalus* polysaccharide	*Astragalus radix*	Glucose, arabinose, galactose, rhamnose and xylose	Periodontitis rat	Rat	Inhibit RANKL expression, promote OPG expression, and reduce TOS and OSI levels	[74]
*Astragalus* polysaccharide	*Astragalus radix*	Glucose, arabinose, galactose, rhamnose and xylose	Osteoclastic precursor cells, periodontitis rat	Rat	Decreased RANKL expression, down‐regulate the expression of RANK, TRAP, and TRAF6, and up‐regulate the level of IL‐10	[75]
Cashew Gum polysaccharide	Cashew nuts	Galactose, glucose, arabinose, rhamnose and glucuronic acid	Periodontitis rats	Rat	Reduce the relative mRNA expression of RANKL and decrease the activity of myeloperoxidase in gingival tissues	[38]
*Morinda officinalis* How polysaccharides	*Morinda officinalis* How	Rhamnose, arabinose, xylose, mannose, glucose, fructose, galactose, galacturonic acid	Root resorption during tooth movement model	Rat	Suppress the expression of ODF and osteoclasts	[76]
*Panax notoginseng* polysaccharide and PNPSⅠ‐Ⅱ	*Panax notoginseng*	Not specified in the literature	Periodontal stem cells	Homo	Lack mechanistic study	[77]
*Panax notoginseng* polysaccharide and PNPSⅠ‐Ⅱ	*Panax notoginseng*	Not specified in the literature	Osteoblast	Mice	Lack mechanistic study	[77]
*Astragalus* polysaccharide	*Astragalus radix*	Glucose, arabinose, galactose, rhamnose, and xylose	Osteoblasts	Rat	Lack mechanistic study	[78]
*Astragalus* polysaccharide	*Astragalus radix*	Glucose, arabinose, galactose, rhamnose, and xylose	Periodontal ligament cells	Homo	Lack mechanistic study	[79]
*Astragalus* polysaccharide	*Astragalus radix*	Glucose, arabinose, galactose, rhamnose, and xylose	Periodontal stem cells	Homo	Up‐regulate miR‐375 and the expressions of Runx‐2, OCN, and OPN	[34]
*Astragalus* polysaccharide	*Astragalus radix*	Glucose, arabinose, galactose, rhamnose, and xylose	Retention stage after orthodontic tooth movement	Rat	Enhance the expression of BMP‐2	[80]
Fucoidan	Brown algae	L‐fucose and sulfate groups	Periodontal stem cells	Homo	Regulate PI3K/Akt and Wnt/*β*‐catenin pathways	[81]
Crude polysaccharides from *Eucommia ulmoides* Oliver	*Eucommia ulmoides* Oliver cortex	Not specified in the literature	Orthodontic tooth movement model	Rat	Promote the expression of Runx2 and Osterix in alveolar bone tissue	[82]

Bone resorption is a pivotal process in periodontal bone remodeling and is predominantly driven by inflammatory responses and osteoclast activity. Multiple plant‐ and fungus‐derived polysaccharides have demonstrated the capability to suppress this process via various mechanisms. For example, Changbai Mountain *Ganoderma lucidum* polysaccharide [31], *Armillaria mellea* polysaccharide [73], konjac glucomannan [62], and CG‐P [38] have all been shown to inhibit alveolar bone resorption in periodontitis models, mainly by modulating inflammation and reducing osteoclast activity. Notably, Changbai Mountain *Ganoderma lucidum* polysaccharide exerts its effects through a unique mechanism of inhibiting macrophage migration in inflamed tissues. This action restricts the dissemination of inflammation and prevents excessive infiltration of immune cells, consequently reducing bone resorption [37].

Moreover, *Astragalus* polysaccharide plays a role in regulating oxidative stress in periodontitis rats. It maintains serum total oxidant status and oxidative stress index at normal levels, effectively alleviating inflammation. Additionally, *Astragalus* polysaccharide influences the immune response by increasing the proportion of CD4^+^Foxp3^+^ regulatory T cells and CD4^+^IL‐10^+^ anti‐inflammatory cells. This modulation helps to create a healthy immune microenvironment, enhancing pathogen clearance, preventing excessive inflammation, and promoting the regeneration of periodontal tissues [74, 75, 83].

Collectively, these findings indicate that the immunoregulatory function of polysaccharides contributes significantly to bone preservation in periodontitis. At the molecular level, this effect is closely associated with the modulation of osteoclast‐related signaling pathways, especially the RANKL–RANK–OPG axis [84]. Under normal conditions, the equilibrium between RANKL and OPG ensures bone homeostasis. However, inflammatory stimuli can disrupt this balance, leading to enhanced osteoclastogenesis and bone resorption. Cashew gum‐derived polysaccharides have been found to downregulate RANKL expression and decrease the RANKL/OPG ratio in periodontal tissues. Simultaneously, it reduces myeloperoxidase activity, thereby inhibiting bone resorption [38].

Similarly, *Astragalus* polysaccharide suppresses the expression of key molecules in the RANKL signaling cascade, such as RANK, tartrate‐resistant acid phosphatase, and TNF receptor‐associated factor 6, and inhibits the activation of MAPK pathways (extracellular signal‐regulated kinase [ERK], c‐Jun N‐terminal kinase, and p38), ultimately resulting in a reduction of osteoclast formation and bone resorption [74, 75, 83].

In addition to modulating inflammatory pathways, some polysaccharides exert anti‐resorptive effects through inflammation‐independent mechanisms. For example, *Morinda officinalis* polysaccharide has been shown to reduce bone resorption in orthodontic tooth movement models [76]. It downregulates the expression of osteoclast differentiation factor and restricts odontoclast activity, indicating that *Morinda officinalis* polysaccharide regulates bone resorption through direct action on osteoclasts.

Beyond the suppression of bone resorption, the promotion of new bone formation is an overarching objective of periodontitis treatment. This complex biological process depends on the precisely coordinated actions of diverse cell populations and molecular signaling cascades, where even minor disruptions can impede tissue repair.

Plant and fungal polysaccharides have emerged as potent regulators of multiple aspects of bone regeneration. These natural macromolecules enhance the proliferation of mesenchymal stem cells (MSCs), drive the osteogenic differentiation of PDLSCs, and facilitate the maturation of osteoblasts, thereby accelerating bone matrix synthesis and mineralization. The underlying mechanisms involve the intricate modulation of intracellular signaling pathways and gene regulatory networks. For example, *Panax notoginseng* polysaccharide, *Astragalus* polysaccharide, and fucoidan activate canonical osteogenic pathways such as phosphatidylinositol 3‐kinase (PI3K)/protein kinase B (PKB/AKT) and Wnt/*β*‐catenin [77–79, 81, 85]. The activation of these pathways upregulates key transcription factors, promoting the expression of bone‐specific proteins essential for matrix deposition. Polysaccharides derived from *Eucommia ulmoides* cortex engage the ERK/BMP‐2/Smad axis, elevating the expression of Runx2 and Osterix to improve alveolar bone microarchitecture in osteoporotic models [82]. *Astragalus* polysaccharide further extends its influence through post‐transcriptional regulation, modulating miRNA networks to upregulate miR‐375 and BMP‐2, thus enhancing the osteogenic differentiation of PDLSCs and osteoblasts [34, 80].

The biological efficacy of polysaccharides is inherently linked to their structural features. Among fucoidan variants, the low‐molecular‐weight form demonstrates superior osteogenic activity in MSCs [85]. This enhanced potency can be attributed to improved solubility, increased cellular uptake, and elevated bioavailability, which enable more efficient interactions with cell surface receptors and robust activation of downstream signaling pathways [86, 87].

The osteogenic properties of these polysaccharides have spurred innovation in the development of biomaterials for bone defect repair. Composite scaffolds, such as fucoidan‐chitosan matrices, *Astragalus* polysaccharide/chitosan/polylactic acid composites, and *Bletilla striata* glucomannan‐based nanostructures [88–92], have excellent biocompatibility with the ability to promote alkaline phosphatase activity, enhanced mineral deposition, and accelerated new bone formation.

In summary, plant and fungal polysaccharides represent a promising class of multifunctional biomolecules for use in periodontal bone regeneration. By regulating osteoclast differentiation, modulating key osteogenic pathways, (including RANKL/OPG, PI3K/Akt, Wnt/*β*‐catenin, and BMP/Smad), and stimulating stem cell‐driven bone formation, they offer a holistic approach to restoring bone homeostasis. Their dual utility as therapeutic agents and biomaterial components, coupled with favorable biocompatibility, makes them valuable candidates for future clinical applications in periodontal bone defect treatment. However, further research is needed to optimize their formulations, elucidate structure–activity relationships, and validate their long‐term safety and efficacy.

## 4. Discussion

**Figure 3:**
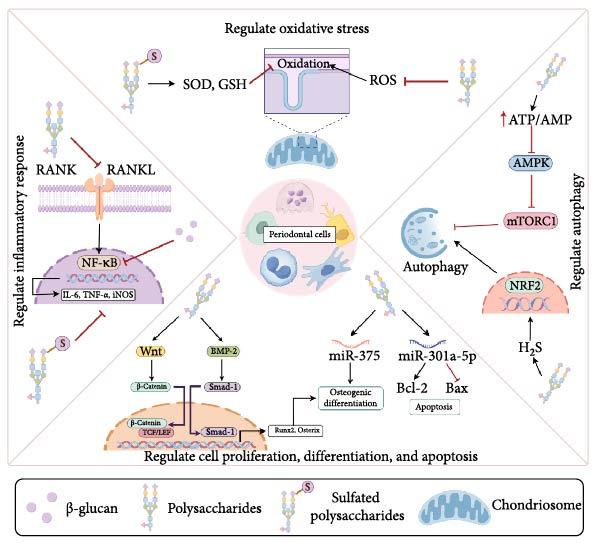
The mechanisms of polysaccharide regulation of periodontitis: As shown above, some of the compounds have multiple signaling targets. AMP, adenosine monophosphate; AMPK, AMP‐activated protein kinase; ATP, adenosine triphosphate; Bax, Bcl‐2‐associated X protein; Bcl‐2, B‐cell lymphoma 2; *β*‐Catenin, beta‐catenin; BMP‐2, bone morphogenetic protein 2; ERK, extracellular signal‐regulated kinases; GSH, glutathione; H_2_S/NRF2 signal, hydrogen sulfide/NRF2 signal; IL‐6, interleukin‐6; iNOS, inducible nitric oxide synthase; miRNA, microRNA; mTORC1, mammalian target of rapamycin complex 1; NF‐*κ*B, nuclear factor kappa‐light‐chain‐enhancer of activated B cells; NRF2, nuclear factor erythroid 2‐related factor 2; PI3/Akt, phosphoinositide 3‐kinase/protein kinase B; RANK, receptor activator of nuclear factor kappa‐B; RANKL, receptor activator of nuclear factor kappa‐B ligand; ROS, reactive oxygen species; Smad‐1, SMAD family member 1; SOD, superoxide dismutase; TCF/LEF, T‐cell factor/lymphoid enhancer‐binding factor; TNF‐*α*, tumor necrosis factor alpha; Wnt, wingless‐related integration site (a family of signaling pathways).

**Figure 4:**
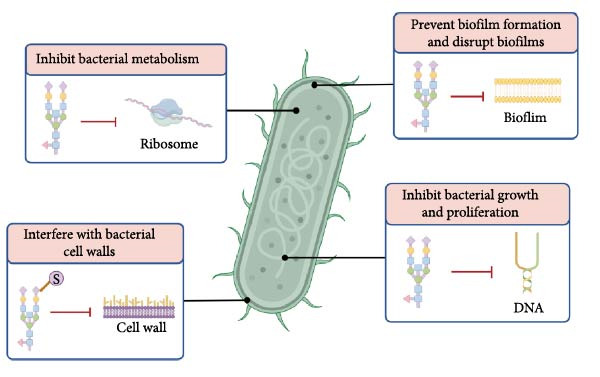
The mechanism of polysaccharide against periodontal pathogenic bacteria.

Accumulating evidence underscores the therapeutic potential of plant and fungal polysaccharides in the management of periodontitis. Preclinical and experimental studies have consistently demonstrated that these polysaccharides can reduce inflammation, suppress pathogenic biofilm formation, and promote periodontal tissue regeneration (Figures 3 and 4). These multifaceted functions highlight their broad therapeutic potential. Among these mechanisms, oxidative stress has emerged as a pivotal contributor to periodontitis initiation and progression. Recent evidence has highlighted the pivotal role of oxidative stress in the initiation and progression of periodontitis. Excessive production of ROS disrupts the local redox balance, contributing to periodontal tissue destruction and the amplification of inflammatory cascades. Meta‐analytic data confirm that patients with periodontitis exhibit significantly elevated total oxidative stress and decreased total antioxidant capacity in the serum, saliva, and gingival crevicular fluid, reflecting a systemic and local redox imbalance that correlates with disease severity [93]. Furthermore, experimental studies have demonstrated that the targeted modulation of oxidative stress, such as through natural compounds like sinensetin, can attenuate ROS‐induced periodontal damage and promote antioxidant responses via molecular mechanisms involving TB and CNC homology 1/Heme oxygenase‐1 signaling [94]. Similar antioxidant and immunomodulatory capacities have been reported for plant‐ and fungal‐derived polysaccharides, suggesting that their ability to restore redox homeostasis may be a key mechanism that complements their anti‐inflammatory, antimicrobial, and regenerative functions. This integrated action further supports their potential as multi‐target agents in periodontitis therapy. Despite their anti‐inflammatory, antioxidant, and osteogenic properties, translation of these natural compounds into clinical practice faces several formidable challenges.

First, the natural origin of polysaccharides inherently introduces variability. Differences in botanical/fungal sources, extraction techniques (e.g., hot water vs. enzymatic extraction), and purification methods (e.g., column chromatography or dialysis) lead to significant batch‐to‐batch variations in composition and purity [95]. Moreover, storage conditions can exacerbate these issues; exposure to humidity may cause viscosity changes, while temperature fluctuations can trigger structural degradation, ultimately compromising product consistency and efficacy.

Second, the structural complexity of polysaccharides is a major hurdle. Although diverse monosaccharide units are linked by various glycosidic bonds in linear or branched configurations, their heterogeneity in terms of molecular weight, branching patterns, and functional group modifications remains poorly understood. Although existing studies have identified key structure–activity relationships, such as the role of moderate molecular weight in enhancing bioactivity or uronic acid content in potentiating anti‐inflammatory effects, precise structural elucidation is lacking [96–104]. Without detailed knowledge of how specific structural features govern biological functions, optimizing polysaccharide‐based therapies for targeted applications remains challenging.

Third, there are significant gaps in understanding the molecular mechanisms underlying the therapeutic effects of polysaccharides. Although their anti‐inflammatory, antioxidant, and osteogenic activities have been observed, the precise signaling pathways and molecular targets involved remain elusive. Most current research focuses on phenotypic outcomes (e.g., reduced inflammation or increased bone formation), overlooking intricate upstream regulatory networks and downstream molecular events. Elucidating these mechanisms is crucial for rational drug design and maximizing therapeutic efficacy.

Fourth, robust clinical validation of safety and efficacy is urgently needed. Although polysaccharides are generally regarded as safe because of their natural origin, individual variability in immune responses and genetic factors can lead to adverse reactions ranging from mild gastrointestinal discomfort to severe allergic responses. Moreover, the limited number of large‐scale randomized clinical trials hinders definitive conclusions regarding the treatment outcomes across diverse patient populations. Establishing the biocompatibility and therapeutic reliability of polysaccharides through rigorous clinical studies is essential for their regulatory approval and widespread adoption.

Finally, challenges related to bioavailability, delivery, and reproducibility impede clinical translation. In the oral cavity, polysaccharides are degraded by oral enzymes and undergo pH‐induced structural changes, thereby reducing their effectiveness. The development of targeted delivery systems, such as nanoparticles or hydrogels, that protect polysaccharides from degradation and enhance their local retention is critical. Additionally, standardizing the manufacturing processes to ensure batch‐to‐batch consistency is essential for reproducible clinical results.

To overcome these barriers, future research should prioritize several key areas. First, the development of standardized extraction, purification, and characterization protocols is imperative for improving product reproducibility and comparability. Second, advanced analytical techniques such as nuclear magnetic resonance spectroscopy and mass spectrometry should be employed to decipher the structure‐function relationships of polysaccharides. Third, comprehensive mechanistic studies, including omics‐based approaches to map signaling networks, are needed to clarify their molecular modes of action. Finally, large‐scale preclinical and clinical trials should be conducted to rigorously evaluate their safety and efficacy, paving the way for the development of polysaccharide‐based therapies as viable alternatives for the treatment of periodontitis.

## 5. Conclusion

As discussed above, plant‐ and fungal‐derived polysaccharides hold significant therapeutic promise for the management of periodontitis, owing to their multifaceted activities, including immunomodulatory, antioxidant, antibacterial, and osteo‐regenerative effects. Preclinical studies have demonstrated that these compounds can reduce inflammation, inhibit pathogenic biofilms, promote tissue regeneration, and counteract oxidative stress, highlighting their potential as host‐modulatory agents for improved periodontal outcomes.

Despite these encouraging findings, several key barriers hinder the clinical translation of polysaccharides. The structural complexity of polysaccharides, limited mechanistic understanding, and lack of standardized extraction and characterization methods have hindered further progress. Moreover, robust in vivo and clinical trials are urgently needed to confirm their safety, optimize delivery strategies, and establish therapeutic efficacy in humans.

In summary, a multidisciplinary effort integrating advanced chemistry, molecular biology, and clinical sciences is critical to bridge these gaps. By addressing these challenges, polysaccharide‐based interventions can evolve into effective, safe, and innovative therapeutics, offering new hope for patients and advancing strategies for the management of periodontitis.

## Conflicts of Interest

The authors declare no conflicts of interest.

## Author Contributions

Y.S. and J.C. contributed to the conception and logic of the article. F.W., S.L., and J.C. contributed to the writing and drafting of the manuscript. Y.S. and J.C. contributed to the critical revision of the manuscript for important intellectual content. All the authors reviewed the manuscript.

## Funding

This work was supported by the funding of Open Project of Guangdong Key Laboratory of Stomatology (Grant number KF2023120104), Student Innovation Capacity Enhancement Program of Guangzhou Medical University, China (Grant number [2024] No. 61‐63), Ministry of Education’s Industry‐University Cooperation Collaborative Education Program, China (Grant number 230718183307236), and National Innovation Training Program for College Students (Grant number 202510570030).

## Data Availability

Data sharing not applicable to this article as no datasets were generated or analyzed during the current study.
